# Clinical value of whole-body PET/CT in patients with active rheumatic diseases

**DOI:** 10.1186/s13075-014-0423-2

**Published:** 2014-08-22

**Authors:** Hiroyuki Yamashita, Kazuo Kubota, Akio Mimori

**Affiliations:** Division of Rheumatic Diseases, National Center for Global Health and Medicine, 1-21-1, Toyama Shinjuku-ku, Tokyo 162-8655 Japan; Department of Radiology, National Center for Global Health and Medicine, 1-21-1, Toyama Shinjuku-ku, Tokyo 162-8655 Japan

## Abstract

Advanced imaging techniques may enable early diagnosis and monitoring of therapy in various rheumatic diseases. To prevent irreversible tissue damage, inflammatory rheumatic disease must be diagnosed and treated in pre-clinical stages, requiring highly sensitive detection techniques. Positron emission tomography (PET) provides highly sensitive, quantitative imaging at a molecular level, revealing the important pathophysiological processes underlying inflammation. This review provides an overview of the current utility of ^18^ F-fluorodeoxyglucose (FDG)-PET/computed tomography (CT) in patients with active rheumatic diseases such as rheumatoid arthritis, spondyloarthritis, polymyalgia rheumatica, adult-onset Still’s disease, relapsing polychondritis, immunoglobulin G4-related disease, large-vessel vasculitis, Wegener’s granulomatosis, polymyositis, and dermatomyositis. We also discuss the role of FDG-PET/CT in the diagnosis and monitoring of these diseases.

## Introduction

Timely diagnosis and early effective treatment can improve the outcome of various inflammatory rheumatic diseases [1]. To enable early diagnosis and individualized therapeutic protocols, sensitive monitoring tools such as advanced imaging techniques are needed. Promising results have already been obtained by using anatomical imaging modalities, such as magnetic resonance imaging (MRI) and ultrasound (US), which allow highly sensitive detection of synovitis and bone marrow edema in inflammatory arthropathies and vascular thickening in systemic vasculitis [2-4]. Each technique, however, has drawbacks and limitations; MRI usually produces images within a limited field of view, and US is limited by variability and labor intensity. In addition, in the presence of inflammation, both techniques can visualize indirect inflammatory signs such as increased tissue water content and hyperperfusion.

Because diagnosis and assessment of disease activity at subclinical stages are increasingly important, nuclear imaging techniques are becoming more widely used. In this review, the potential role of ^18^ F-fluorodeoxyglucose positron emission tomography/computed tomography (FDG-PET/CT) in the diagnosis and monitoring of inflammatory rheumatic diseases is discussed.

## Part I. The usefulness of PET/CT imaging in the assessment of the severity of the disease

### FDG-PET for inflammatory diseases

Increased ‘aerobic glycolysis’ in cancer cells, originally described by Otto Warburg, provides a growth advantage to tumor cells. With the imbalance in the growth of capillaries and cancer cells, cancer cells become hypoxic. The transcription factor hypoxia-inducible factor 1α (HIF1α) mediates cancer cell metabolism and shifts cancer cells into oxygen conservation mode, aerobic glycolysis, so that the reduced oxygen consumption saves cancer cells from anoxic death; that is, HIF1α regulates the expression of glucose transporters, hexokinase, and other factors in cancer cells [5].

FDG is used to trace glucose metabolism. Many cancer cells showed elevated expression of glucose transporters and hexokinase. Most cancer cells are FDG-avid, and a fusion imaging technique combining PET/CT, which provides information on both anatomy and glucose metabolism, has improved the diagnostic accuracy and is now widely used in oncology.

FDG uptake is not limited to cancer cells; uptake may also occur in various inflammatory cells. Elevated FDG uptake by activated macrophages and by newly formed granulation tissue was demonstrated by Kubota and colleagues [6,7] in the early 1990s. The uptake of FDG by cancer cells is postulated to involve the same mechanism as in inflammatory cells. Cramer and colleagues [8] reported that HIF1α activation is essential for myeloid cell (granulocytes and monocytes/macrophages) infiltration and activation an *in vivo* inflammation model. More recently, Matsui and colleagues [9] reported FDG uptake in the area in which inflammatory cell infiltration and synovial cell hyperplasia were visible in an arthritis model. Based on *in vitro* experiments, Matsui and colleagues suggested that the cell types responsible for FDG uptake are activated macrophages and proliferating fibroblasts in the presence of cytokine stimulation and under hypoxic circumstances within a joint. The FDG uptake by inflammatory tissue, such as arthritis lesions, seems to reflect the inflammatory activity accurately. Such studies have strongly encouraged the clinical application of FDG-PET/CT for rheumatic diseases.

### Rheumatoid arthritis

**Figure 1:**
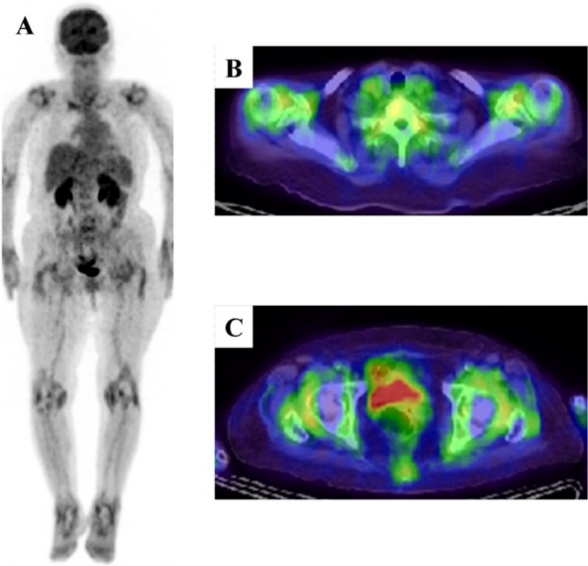
**Typical** ^**18**^ **F-fluorodeoxyglucose-positron emission tomography/computed tomography (FDG-PET/CT) findings in a patient with rheumatoid arthritis (RA). (A)** Maximum intensity projection and **(B, C)** axial FDG-PET/CT findings of RA in an 86-year-old woman presenting with polyarthritis. She was diagnosed with RA based on the presence of symmetrical polyarthritis that continued for more than 19 weeks with a severe inflammatory reaction (C-reactive protein, 12.72 mg/dL) and magnetic resonance imaging findings that showed synovitis and tendinitis. FDG-PET/CT showed **(A)** symmetrical arthritis and remarkable circular FDG uptake due to synovitis around the **(B)** shoulders and **(C)** hips.

Typical FDG-PET/CT images of rheumatoid arthritis (RA) are shown in Figure 1. In 1995, the first FDG-PET studies in patients with active RA revealed increased FDG uptake in clinically inflamed wrist joints. Furthermore, standardized uptake values (SUVs) for FDG were correlated with clinical indicators such as tenderness and swelling [10]; these findings were confirmed in other studies [11,12]. FDG SUV data in arthritis are also correlated with disease activity score 28 (DAS28) and simple disease activity index values. The number of FDG-positive joints is also strongly correlated with their cumulative SUV and disease duration [11]. However, Goerres and colleagues [13] found that simple visual semi-quantitative scoring (from 0 to 4) based on FDG joint uptake [12] also reflected clinical evidence of inflammation in joints. This approach eliminates the need to quantify tracer uptake but lacks objectivity. On the other hand, the semi-quantitative analysis using SUVs has better objectivity. Okamura and colleagues [14] found that the SUVs for 12 joints correlated with tender joint count (TJC) and that the SUVs for eight joints correlated with DAS28, DAS28-C-reactive protein (CRP), and TJC in patients with RA, suggesting a close relationship between SUVs for large joints and disease activity.

Beckers and colleagues [11] reported sensitivities of up to 90% in a study evaluating 356 joints of 21 patients with established RA by using FDG-PET. In this study, visually identified FDG positivity was clearly associated, according to odds ratios, with both joint swelling and tenderness. Regarding specificity, FDG-PET allows excellent differentiation between inflamed and healthy joints, both among patients with RA and between patients and healthy controls; healthy joints do not take up the tracer at all [11,15]. However, despite a greater number of FDG-positive joints in RA than in osteoarthritis patients, absolute values for tracer uptake do not differ between these two conditions [16]. Nonetheless, whole-body PET may aid in differentiation between RA and other inflammatory joint diseases, as differences in bio-distribution patterns have allowed distinction between some forms of arthritis associated with connective tissue disease [17].

Recently, several studies have indicated that subclinical disease is present during clinical remission and may be related to progression of joint damage [18]. Owing to its high sensitivity and ability to scan multiple joints in one session, PET may allow detection of such subclinical disease activity. Whole-body PET scanning in 18 patients with RA, four of whom were in remission, showed significant differences between those with active arthritis and those in clinical remission [12]. FDG uptake by large joints, total visual PET scores for involved joints, SUV_max_, and the mean number of joints per patient with high FDG uptake were all significantly lower for patients in clinical remission. In all patients in remission, however, increased FDG uptake was still observed in one or more joints, suggesting subclinical disease activity.

Besides its ability to detect and monitor subclinical disease, PET may have prognostic power. Recently, FDG changes in inflamed hand joints after 2 weeks of infliximab treatment were correlated with DAS28 joint scores at 14 to 22 weeks of treatment [19]. In contrast, erythrocyte sedimentation rate (ESR), CRP, and DAS28 scores had no such predictive value after 2 weeks of therapy.

Comparison of semiquantitative PET data with US data and clinical findings [11] revealed a significant linear correlation between SUVs and synovial thickness, as measured by US, for nearly all joints. This relationship was stronger for larger joints, as these are more accurately evaluated by semiquantitative scoring [20]. Moreover, for anatomical reasons, some small joints in the hand cannot easily be evaluated in three dimensions by US [21]. The first study simultaneously investigating PET, MRI, and US in patients with RA showed that enhanced FDG uptake was associated with positive findings obtained by the other two imaging techniques [16]. In addition, the study revealed correlations among SUVs from PET, relative contrast enhancement from MRI, and synovial thickness from US.

Over the last few years, hybrid PET-CT and PET-MRI have become available. Combining PET with CT has helped place pathophysiologic PET information in its anatomic context [22]. Thus, hybrid imaging should allow more precise localization of PET signals, and PET-MRI should limit radiation exposure [23].

### Spondyloarthritis

**Figure 2:**
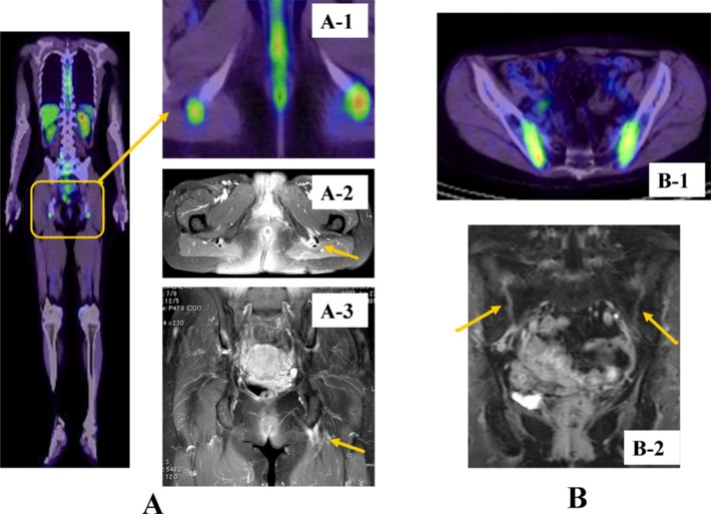
**Typical** ^**18**^ **F-fluorodeoxyglucose-positron emission tomography/computed tomography (FDG-PET/CT) and magnetic resonance imaging (MRI) findings in patients with spondyloarthritis (SpA), enthesitis of the ischial tuberosity, and sacroiliitis. (A)** Whole-body FDG-PET/CT in a patient with SpA reveals FDG accumulation in the ischial tuberosity (A1). MRI (fat-suppressed, T1-weighted imaging) reveals enhancement in the areas surrounding the ischial tuberosity, consistent with enthesitis (A2-3). **(B)** FDG accumulation suggestive of sacroiliitis in the sacroiliac joint in patients 1 and 2 on FDG-PET/CT (B1). MRI (fat-suppressed, T1-weighted imaging) identifies contrast enhancement in patient 2 (B2).

Typical FDG-PET/CT images of spondyloarthritis (SpA) are shown in Figure 2 [24]. SpA includes ankylosing spondylitis (AS), psoriatic arthritis (PsA), reactive arthritis, enteropathic arthritis, and undifferentiated SpA [25]. SpA often involves enthesitis, sacroiliitis, and inflammatory spondylitis [26].

Taniguchi and colleagues [27] evaluated the accuracy of FDG-PET/CT in detecting enthesitis in patients with SpA. PET/CT scans of the shoulder, hip, and knee joints revealed that FDG accumulates at the entheses in SpA and in the synovium in patients with RA. SUV_max_ was significantly higher at the enthesis of the lumbar spinous process, pubic symphysis, and ischial tuberosity in patients with SpA than in patients with RA. Lumbar spinous processes and ischial tuberosities appeared more frequently via PET/CT than MRI in patients with SpA. The authors concluded that PET/CT represents an alternative modality to identifying enthesitis and will likely contribute to the early diagnosis of SpA.

Strobel and colleagues [28] evaluated the performance of FDG-PET/CT for the diagnosis of sacroiliac joint (SIJ) arthritis in patients with active AS by using patients with mechanical low back pain (MLBP) as a control. The mean ratios of FDG uptake in the SIJ to that in the sacrum were 1.66 in patients with AS and 1.12 in patients with MLBP. With plain radiography as the gold standard and using a sacroiliac joint to sacrum (SIJ/S) ratio of 1.3 as the threshold, the sensitivity and specificity of FDG-PET/CT for arthritis were 80% and 77%, respectively. On a per-SIJ basis, the greatest sensitivity (94%) was found in grade 3 sacroiliitis. These results suggest that quantitative FDG-PET/CT may be useful to diagnose sacroiliitis in active AS, providing an alternative to conventional bone scintigraphy in times of molybdenum shortage.

We used FDG-PET/CT to compare SUVs in various joints in 53 patients with SpA, polymyalgia rheumatica (PMR), and RA [24]. In patients with SpA, SUV_max_ in the SIJ was greater than in patients with PMR or RA. No significant difference in vertebral scores was observed among groups. PET/CT findings thus can distinguish SpA from RA and PMR and are useful for the early diagnosis of sacroiliitis.

Vijayant and colleagues [29] evaluated the potential of FDG-PET in the early assessment of treatment response in various rheumatic diseases, including 11 patients with newly diagnosed SpA and one patient with PsA. In the SpA group, FDG uptake in the affected joint was heterogeneous, low-grade, and non-symmetrical, with intense tendon and muscular uptake in symptomatic joints. In contrast, FDG uptake in the patient with PsA was intense in the joints and soft tissue. If larger studies corroborate these findings, FDG-PET could be useful to distinguish RA from SpA.

### Polymyalgia rheumatica

**Figure 3:**
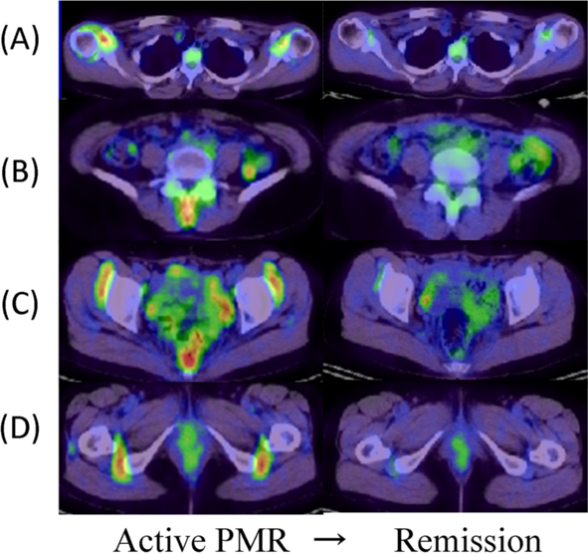
^**18**^ **F-fluorodeoxyglucose-positron emission tomography/computed tomography (FDG-PET/CT) images at diagnosis and after steroid treatment in a patient with isolated polymyalgia rheumatica (PMR).** FDG uptake in the **(A)** shoulders, **(B)** spinous processes of the lower lumbar vertebrae, **(C)** iliopectineal bursitis, and **(D)** ischial tuberosity is normalized after therapy (A-D, right panels).

Typical FDG-PET/CT images of PMR are shown in Figure 3 [30]. PMR is an inflammatory rheumatic disease characterized by aches and morning stiffness in the shoulders, hip girdle, and neck in patients over 50 years of age. PMR is diagnosed by the exclusion of other disorders causing similar complaints and by its rapid response to low-dose corticosteroid therapy [31,32]. Although its pathology is unknown, synovitis and bursitis are common features of this disease. MRI and US frequently reveal inflammation of the tenosynovial sheaths of the hands or feet [33-35].

Moosig and colleagues [36] quantified FDG accumulation in large vessels in 13 untreated patients with PMR by PET and compared these data with serological markers of inflammation. By visual evaluation, FDG uptake by the aorta or its major branches increased in 12 of 13 patients. In active PMR, the mean region of interest (ROI) index for all vascular regions exceeded that of controls by 70%. In active PMR, FDG uptake was significantly correlated with CRP, ESR, and platelet counts. The observed FDG accumulation in the aorta and its branches and the strong correlation between tracer uptake and markers of inflammation suggest that large-vessel arteritis is characteristic of active PMR.

Blockmans and colleagues [37] investigated whether FDG deposition in various vascular lesions and large joints of patients with isolated PMR predicts relapse. All patients underwent an FDG-PET scan before steroid treatment and at 3 and 6 months; seven vascular areas were scored, and a total vascular score (TVS), ranging from 0 to 21, was calculated. At diagnosis, vascular FDG uptake was noted in 31% of patients, predominantly at the subclavian arteries. FDG uptake in the shoulders was noted in 94% of patients, in the hips in 89%, and in the spinous process of the vertebrae in 51%. FDG uptake intensity was not correlated with the risk of relapse in either the large vessels or large joints.

Whereas Blockmans and colleagues [37] analyzed FDG-PET changes in patients with PMR, we analyzed the precise distribution of lesions via PET/CT and evaluated differences in FDG accumulation between PMR and similar diseases. In patients with PMR, FDG uptake was increased in ischial tuberosities, greater trochanters, and lumbar spinous processes [30]. Positive results at two or more of these sites were highly sensitive (85.7%) and specific (88.2%) for the diagnosis of PMR, and shoulder or hip joint involvement was not disease-specific. High FDG accumulation was found in the aortas and subclavian arteries of two PMR patients in whom FDG uptake did not identify temporal arteritis or scanty synovium and perisynovium. PET/CT images of the 12 PMR patients without apparent vascular involvement revealed synovitis or perisynovitis or both.

We also used FDG-PET/CT to compare SUVs in various joints in 53 patients with SpA, PMR, and RA [24]. In patients with PMR, the SUV_max_ for ischial tuberosities was significantly higher than in patients with SpA or RA, and those in the greater trochanter and spinous processes were also significantly higher than in patients with RA.

Furthermore, we compared PET/CT findings in a large number of PMR cases with those in patients with elderly-onset RA (EORA), which is extremely difficult to distinguish from PMR. We observed no significant difference in FDG uptake in the shoulders or hips. However, specific uptake patterns were observed in each group: circular and linear uptake patterns around the humeral head in EORA and focal and nonlinear uptake patterns in PMR. Moreover, focal uptake at the front of the hip joint, indicating iliopectineal bursitis, tended to be limited to the PMR group. The sensitivity and specificity for PMR diagnosis were very high at 92.6% and 90.0%, respectively, when at least three of the five items, including findings characteristic of shoulder and iliopectineal bursitis, FDG uptake in ischial tuberosities and spinal spinous processes, and lack of FDG uptake in the wrists, were satisfied. FDG-PET/CT may be useful for the detection of PMR lesions, which are difficult to identify by using other methods.

### Adult-onset Still’s disease

**Figure 4:**
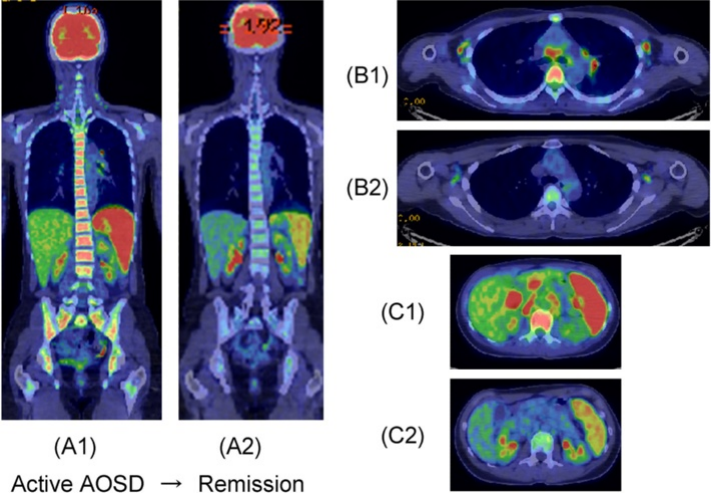
^**18**^ **F-fluorodeoxyglucose-positron emission tomography/computed tomography (FDG-PET/CT) images at diagnosis and after steroid and tocilizumab treatment in a patient with adult-onset Still’s disease (AOSD).** (A1, B1, and C1) Marked FDG accumulation was observed in the bone marrow, spleen, and multiple lymph nodes at diagnosis. (A2, B2, and C2) After treatment, FDG uptake decreased in these sites - bone marrow, from SUV_max_ = 4.02 (A1) to 2.50 (A2); spleen, from SUV_max_ = 6.05 (A1 and C1) to 4.38 (A2 and C2) - as well as in multiple lymph nodes, including in the axilla, mediastinum, hilar region of the lung, hilar region of the liver, and para-aortic region (B1/C1 → B2/C2). SUV, standardized uptake value.

Typical FDG-PET/CT images of adult-onset Still’s disease (AOSD) are shown in Figure 4 [38]. We first evaluated FDG-PET/CT for diagnosis and disease evaluation of AOSD by investigating FDG uptake for characteristic findings in seven patients with AOSD and reviewing the literature on seven previous reports of PET/CT in patients with AOSD [38]. FDG accumulation was positive mainly in the bone marrow (100%), spleen (90.9%), lymph nodes (80.0%), and joints (75.0%). In addition, FDG uptake was positive in the pericardium, pleura, salivary glands, eyelids, muscle, and major blood vessels. Follow-up PET/CT showed diminished FDG accumulation, as measured by SUV_max_, in the bone marrow, spleen, and lymph nodes. The only correlation with laboratory data was between lactate dehydrogenase and spleen SUV. In conclusion, FDG-PET/CT is useful for long-term assessment of AOSD activity in individual patients. However, PET/CT findings alone are not sufficient to make a differential diagnosis of AOSD versus malignant lymphoma.

### Relapsing polychondritis

**Figure 5:**
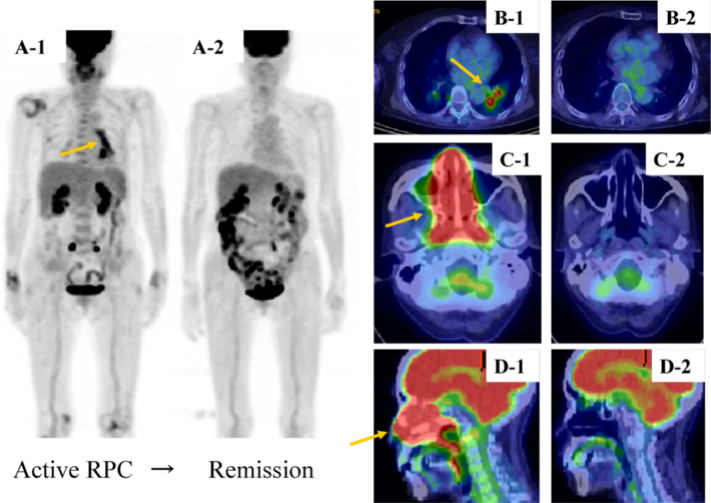
**Comparison of pre- and post-treatment** ^**18**^ **F-fluorodeoxyglucose-positron emission tomography/computed tomography (FDG-PET/CT) images in a patient with relapsing polychondritis (RPC). (A)** Maximum intensity projection and **(B-D)** axial FDG-PET/CT findings of RPC in a 74-year-old female patient presenting with nasal symptoms. The patient was positive for type II anti-collagen antibody, and nasal cartilage biopsy was consistent with RPC. FDG accumulation (SUV_max_ = 13.03) is well defined from the infrahilar region of the left inferior lobe to the pulmonary hilus (A1 and B1, arrow) and conspicuous in the nasal cavity (SUV_max_ = 9.50) (C1 and D1, arrow). Neither bronchial wall thickening nor bronchial stricture is apparent. Post-treatment images show a complete lack of accumulation (A2-D2). SUV, standardized uptake value.

Typical FDG PET/CT images of relapsing polychondritis (RPC) are shown in Figure 5 [39]. RPC is relatively rare, and early diagnosis is difficult. We first investigated the utility of FDG-PET/CT for the diagnosis and evaluation of disease activity in five RPC patients undergoing FDG-PET/CT in our hospital and eight cases in the literature [39]. Typical FDG accumulation was noted in tracheobronchial trees, costal cartilage, joints, larynx, nasal cavity/paranasal sinuses, auricles, lymph nodes, and the aorta. In one patient, PET revealed nasal chondritis despite an absence of nasal changes upon physical examination. Of five patients with costochondritis, four remained asymptomatic. Of nine patients with airway FDG accumulation, eight developed respiratory symptoms and all had CT abnormalities. In the remaining patient, airway FDG accumulation was evident despite the absence of airway symptoms and a lack of abnormalities in the respiratory function test or CT. PET also revealed bronchial chondritis in asymptomatic patients. In the five patients examined by PET post-treatment, FDG accumulation diminished as symptoms improved and inflammation decreased. We conclude that FDG-PET/CT is a potentially powerful tool for the early diagnosis of RPC, especially in patients with affected organs that are difficult to biopsy. This modality also facilitates the evaluation of extent of disease and disease activity during treatment.

### IgG4-related disease

IgG4-related disease (IgG4-RD) is a systemic disorder associated with lesions characterized by mass formation in multiple specific organs. This disorder comprises Mikulicz’s disease, autoimmune pancreatitis (AIP), hypophysitis, Riedel thyroiditis, interstitial pneumonitis, interstitial nephritis, lymphadenopathy, retroperitoneal fibrosis, and inflammatory pseudotumour [40]. The combination of such lesions on FDG-PET/CT may strongly suggest or support the diagnosis of IgG-4RD.

Shigekawa and colleagues [41] noted that the FDG-PET pattern at baseline, including extra-abdominal lymph nodes or salivary glands (or both) and the involvement of the eyes and biliary ducts, can be useful for discriminating between AIP and pancreatic cancer. Ozaki and colleagues [42] also found that FDG uptake by hilar lymph nodes was significantly more frequent in AIP than in pancreatic cancer and reported that uptake by the lacrimal gland, salivary gland, biliary duct, retroperitoneal space, and prostate was seen only in AIP. They reported that a longitudinal pattern, heterogeneous accumulation, and multiple localizations in the pancreas indicated AIP rather than pancreatic cancer [42].

Ebbo and colleagues [43] evaluated FDG-PET/CT for disease staging and treatment evaluation in 46 FDG-PET/CT images from 21 patients with IgG4-RD. At diagnosis or relapse, all patients presented abnormal FDG uptake at sites typically affected by IgG4-RD. In most cases, FDG-PET/CT was more sensitive than conventional imaging to detect organ involvement, especially in the arteries, salivary glands, and lymph nodes. In a few cases, FDG-PET/CT failed to identify small or contiguous lesions in the brain or kidneys.

In addition, we evaluated the utility of FDG-PET/CT in eight patients with IgG4-RD [44]. Although nearly all patients were negative for CRP, various organs took up significant amounts of FDG. In conclusion, FDG accumulation in organs characteristically affected by IgG4-RD allows diagnosis without evidence of an associated inflammatory reaction.

### Large-vessel vasculitis

**Figure 6:**
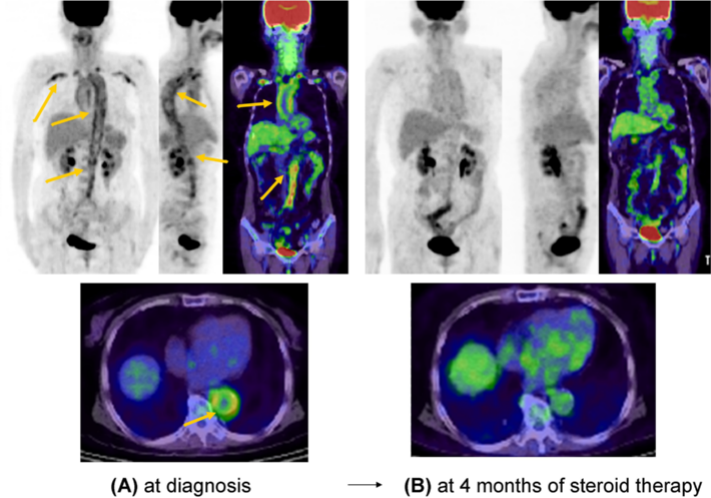
^**18**^ **F-fluorodeoxyglucose-positron emission tomography/computed tomography (FDG-PET/CT) images at diagnosis and after steroid treatment in a patient with large-vessel vasculitis. (A)** FDG-PET/CT at diagnosis identifies aortitis in the thoracic and abdominal aorta and arteritis bilaterally in the subclavian and axillary vessels as strong uptake in the walls of the aorta and arteries. **(B)** Update decreased markedly during steroid treatment.

Typical FDG-PET/CT images of large-vessel vasculitis (LVV) are shown in Figure 6 [45]. From a systematic review on FDG-PET/CT in patients with LVV, Treglia and colleagues [46] drew several conclusions. First, FDG-PET/CT appears to be useful in early diagnosis and in the assessment of disease activity and extent. Second, the correlation between FDG-PET findings and serological inflammatory markers as well as the usefulness of FDG-PET/CT in evaluating treatment response require further investigation. Additionally, FDG-PET/CT appears to be superior to conventional imaging methods, such as US or MRI, in the diagnosis of LVV but not in predicting relapse or evaluating vascular complications. Lastly, PET analysis and diagnostic criteria should be standardized to allow reproducible, directly comparable results.

We also studied the usefulness of FDG-PET/CT and contrast-enhanced CT in early diagnosis and treatment follow-up of patients with LVV presenting as elderly-onset inflammation of unknown origin (IUO) [45]. For quantitative comparison, we evaluated SUV_max_ and PET scores of the aortic wall, as well as aortic wall thickness (W) and its ratio with respect to aortic radius (W/R) by contrast-enhanced CT, and compared pre-treatment and post-treatment values. Of 124 patients who were hospitalized because of advanced age and IUO, 10.5% had LVV, and more than half had non-specific symptoms. Compared with control subjects, patients with LVV showed significantly higher aortic wall SUV_max_, higher PET scores as revealed by FDG-PET/CT, and increased aortic wall thicknesses as revealed by contrast-enhanced CT. In conclusion, LVV is an important cause of IUO with non-specific symptoms in older patients. Imaging examination comprising contrast-enhanced CT and FDG-PET/CT is useful for early diagnosis and early treatment evaluation of LVV, as it detects amelioration of aortic wall thickening.

Notably, FDG-PET/CT may be negative for old lesions even if the arterial stricture is severe. Lesions in which the inflammatory activity has already subsided may not be appropriate for evaluation by FDG-PET; in such cases, morphological imaging such as MRI or contrast-enhanced CT may be used. Again, LVV should be diagnosed in the early phase with FDG-PET/CT to prevent stricture formation.

### Wegener’s granulomatosis

Wegener’s granulomatosis (WG), namely granulomatosis with polyangiitis, is a relatively rare disease characterized by granulomatous necrotizing vasculitis. In the first evaluation of FDG-PET/CT imaging for the diagnosis and monitoring of WG [47], we retrospectively analyzed 13 FDG-PET/CT images obtained from eight patients. WG lesions of the upper respiratory tract and lung were more clearly detected by FDG-PET/CT fusion imaging than by non-enhanced CT alone. In addition, FDG-PET/CT can be combined with other imaging methods to inform selection of biopsy sites. Ozmen and colleagues [48] also reported PET/CT to be useful for detecting active lesions (except in the kidneys) and for identifying biopsy sites in patients with WG. FDG-PET/CT is more useful than traditional imaging studies, such as CT, for differentiating between active lesions due to WG and residual foci of inflammation. It is also useful for detecting active lesions in unexpected sites such as large vessels and the spleen. In conclusion, FDG PET/CT is a feasible modality for evaluating lesion activity in WG.

### Polymyositis and dermatomyositis

Owada and colleagues [49] examined whether FDG-PET can detect myositis or extramuscular lesions in patients with polymyositis (PM) and dermatomyositis (DM) and observed increased FDG uptake in muscle in 33% of patients. The sensitivity of FDG-PET to detect myositis was lower than that of electromyography, MRI, and muscle biopsy, and patients with and without increased FDG uptake in muscle did not differ clinically, although those with FDG muscle uptake had a tendency toward extended myositis with endomysial cell infiltration. In contrast, FDG-PET did detect neoplasms in patients with associated malignancy, which accounted for 38.9% of patients with interstitial lung disease. Thus, FDG-PET imaging appears to have limited usefulness for the evaluation of myositis in patients with PM and DM because of its low sensitivity.

On the other hand, Tanaka and colleagues [50] also conducted a retrospective study to determine whether FDG-PET/CT discriminates PM and DM from non-muscular diseases and whether FDG uptake in proximal muscles reflects severity of muscular inflammation. Mean proximal muscle SUVs were significantly greater in patients with PM and DM than in controls and were correlated with mean proximal manual muscle test scores and serum creatine kinase and aldolase levels. Furthermore, SUVs in proximal muscles from which biopsy specimens were obtained were significantly correlated with histological grade for inflammatory cell infiltration. These results suggest that FDG-PET/CT is useful in the diagnosis of PM and DM when inflammation in proximal muscles is also quantitatively assessed by other means. These results also indicate that local FDG uptake reflects inflammatory activity in proximal muscle and can help guide biopsy site selection.

## Part II. The usefulness of PET/CT imaging in the therapy response assessment

### Rheumatoid arthritis

FDG-PET images do detect reductions in metabolism and treatment-related changes in the volume of pannus that are not detectable with conventional imaging [10]. In 2006, Beckers and colleagues [51] used FDG-PET to image joints before and after anti-TNF therapy and found that PET positivity was correlated with higher SUVs. In contrast, FDG-PET revealed no significant metabolic changes following acupuncture treatment of affected knees in six patients with chronic RA [52].

Recently, Okamura and colleagues [14] investigated the correlations between SUV_max_ in 12 large joints and clinical parameters in 22 patients with RA before and after treatment with biologics. The results showed positive correlations of ΔSUV with ΔDAS28, ΔDAS28-CRP, and ΔTJC in large joints before and after treatment, suggesting the usefulness of PET in evaluating treatment responses [14].

A number of studies have examined the ability of PET to accurately indicate treatment outcomes compared with MRI. Before and during treatment with non-steroidal anti-inflammatory drugs, prednisone, or methotrexate, changes in joint uptake of FDG, as revealed by PET, were strongly correlated with synovial volume upon MRI in patients with RA [10,53].

The first study simultaneously investigating PET, MRI, and US in patients with RA showed that changes in SUVs in RA-affected knees after initiation of anti-TNF therapy were also correlated with changes in MRI parameters and serum CRP and metalloproteinase-3 levels but not with changes in synovial thickness as measured via US [51].

### Spondyloarthritis

Vijayant and colleagues [29] evaluated the potential of FDG-PET in the early assessment of treatment response in various rheumatic diseases, including one patient with PsA. Post-treatment scans were performed in one patient with PsA. There was a significant fall in SUV_max_ correlating to the clinical improvement in the patient. FDG-PET also appears to be a sensitive tool in the early assessment of treatment response, especially when using quantitative information.

### Polymyalgia rheumatica

Typical FDG-PET/CT images of PMR are shown in Figure 3 [24]. In our case, FDG accumulation tended to decrease in the areas of bursitis and iliopsoas bursitis of the shoulder, the spinous process, and the ischial tuberosity after treatment, suggesting the usefulness of FDG-PET/CT for evaluating treatment effects.

Moosig and colleagues [36] quantified FDG accumulation in large vessels in 13 untreated patients with PMR by PET. Among the eight patients who underwent follow-up PET, the mean ROI index for all vascular regions declined substantially.

Blockmans and colleagues [37] investigated whether FDG deposition in various vascular lesions and large joints of patients with PMR predicts relapse. All patients underwent an FDG-PET scan before steroid treatment and at 3 and 6 months. Repetitive PET scintigraphy after 3 months of steroid treatment resulted in a decrease of TVS and a lower intensity of FDG uptake in shoulders, hips, and spinous process. At 3 months, all but two patients had significantly decreased laboratory parameters of inflammation. This decrease of FDG uptake probably reflects a lower disease activity, but sedimentation rate and CRP levels, which are much cheaper to perform, reflect the same. So this does not justify a repeat PET scan.

### Adult-onset Still’s disease

Typical FDG-PET/CT images of AOSD are shown in Figure 4 [38]. In our case, FDG accumulation tended to decrease in the bone marrow, spleen, and lymph nodes after treatment, suggesting the usefulness of FDG-PET/CT for evaluating treatment effects.

### Relapsing polychondritis

Typical FDG PET/CT images of RPC are shown in Figure 5 [39]. In our case, FDG accumulation tended to decrease in the bronchial wall, hilar lymph nodes, and nasal cavity after treatment, suggesting the usefulness of FDG PET/CT for evaluating treatment effects.

### IgG4-related disease

Ebbo and colleagues [43] evaluated FDG-PET/CT for disease staging and treatment evaluation in 46 FDG-PET/CT images from 21 patients with IgG4-RD. Evaluation before and after treatment showed that FDG-PET/CT results were generally correlated with treatment response and disease activity. This retrospective study shows that FDG-PET/CT imaging is useful for staging IgG4-RD and assessing treatment response. In addition, residual organic pathology may be present even if serum IgG4 levels have been decreased by steroid therapy. In such cases, PET images are considered to be useful for evaluating the IgG4-RD disease activity.

### Large-vessel vasculitis

Typical FDG-PET/CT images of LVV are shown in Figure 6 [45]. In our case, FDG accumulation tended to decrease in the thoracoabdominal aortic aorta and bilateral subclavian and axillary arteries, suggesting the usefulness of FDG-PET/CT for evaluating treatment effects.

From a systematic review on FDG-PET/CT in patients with LVV, Treglia and colleagues [46] concluded that FDG-PET/CT appears to be superior to conventional imaging methods in assessing response to immunosuppressive treatment.

We also studied the usefulness of FDG-PET/CT and contrast-enhanced CT in early diagnosis and treatment follow-up of patients with LVV [45]. PET scores and contrast-enhanced CT revealed significant reductions in aortic wall thickness following treatment. In conclusion, imaging examination comprising contrast-enhanced CT and FDG-PET/CT is useful for early treatment evaluation of LVV, as it detects amelioration of aortic wall thickening.

### Wegener’s granulomatosis

We retrospectively analyzed 13 FDG-PET/CT images obtained from eight patients with WG. All active lesions showed decreased FDG uptake after treatment [47]. Ozmen and colleagues [48] also showed significantly decreased FDG uptake after complete remission of WG in their PET/CT study. FDG-PET/CT is more useful than traditional imaging studies, such as CT, for differentiating between active lesions and residual loci of inflammation in patients with WG. Furthermore, PET/CT allows earlier evaluation of treatment responses as well as earlier detection of recurrent disease than CT. In conclusion, FDG PET/CT is a feasible modality for evaluating therapeutic efficacy in WG.

## The potential applications of immuno-PET

The development of non-invasive imaging techniques using monoclonal antibodies (mAbs) is a quickly evolving field. Immuno-PET uses positron-emitting isotopes to track the localization of mAbs with excellent image quality. Procedures for labeling mAbs with ^89^Zr or ^124^I by using good manufacturing procedures have been established, and therefore these radiopharmaceuticals are being investigated for clinical use. Immuno-PET is expected to become an option as a non-invasive diagnostic tool providing ‘comprehensive immunohistochemical staining *in vivo*’ [54]. Therefore, immuno-PET has enormous potential for diagnosing rheumatic disease and evaluating its activity in the presence of disease-specific mAbs.

## Summary of review

It is sometimes difficult to distinguish between RA and other rheumatic diseases such as SpA and PMR, which lack specific serum markers; PET may be useful for differentiating among these conditions. Moreover, no specific serum markers exist for AOSD, making its differentiation from other rheumatic diseases occasionally difficult. However, the finding of FDG accumulation in the bone marrow, spleen, lymph nodes, and joints in this condition is useful. It should be acknowledged, however, that differentiation of AOSD from malignant lymphomas is sometimes difficult. As for RPC, it is possible to identify asymptomatic chondritis by using PET and readily identify the distribution of the lesions. With respect to IgG4-RD, the diagnostic yield can be increased by recognizing the characteristic distribution of the lesions in this disease by using PET combined with the absence of a significant inflammatory response. In addition, LVV can be cited in the differential diagnosis as a cause of IUO in older patients who present with non-specific symptoms only; PET is useful for early diagnosis of this condition, and early therapeutic intervention may prevent stenotic lesions. With regard to WG (granulomatosis with polyangiitis (GPA)), PET is useful for the evaluation of active lesions, therapeutic monitoring, and identification of biopsy sites. As for inflammatory myopathy, whereas one report suggests the limited usefulness of PET because of its low sensitivity, another report suggests that PET provides useful information for determining the appropriate biopsy sites.

## Conclusions

FDG-PET/CT can provide information on active inflammatory lesions. It is a very sensitive but non-specific imaging modality. The distribution patterns of inflammatory foci sometimes suggest specific disease. In general, if FDG-PET/CT imaging is used appropriately, it may provide very helpful information for accurate diagnosis. In addition, FDG-PET/CT is very sensitive for monitoring disease activity. It could be applied to the prediction of therapeutic response, but further studies are required. FDG-PET/CT is a promising imaging modality for rheumatic diseases.

## Abbreviations

AIP: Autoimmune pancreatitis
AOSD: Adult-onset Still’s disease
AS: Ankylosing spondylitis
CRP: C-reactive protein
CT: Computed tomography
DAS28: Disease activity score 28
DM: Dermatomyositis
EORA: Elderly-onset rheumatoid arthritis
ESR: Erythrocyte sedimentation rate
FDG: ^18^ F-fluorodeoxyglucose
HIF1α: Hypoxia-inducible factor 1α
IgG4-RD: Immunoglobulin G4-related disease
IUO: Inflammation of unknown origin
LVV: Large-vessel vasculitis
mAb: Monoclonal antibody
MLBP: Mechanical low back pain
MRI: Magnetic resonance imaging
PET: Positron emission tomography
PM: Polymyositis
PMR: Polymyalgia rheumatica
PsA: Psoriatic arthritis
RA: Rheumatoid arthritis
ROI: Region of interest
RPC: Relapsing polychondritis
SIJ: Sacroiliac joint
SpA: Spondyloarthritis
SUV: Standardized uptake value
TJC: Tender joint count
TNF: Tumor necrosis factor
TVS: Total vascular score
US: Ultrasound
WG: Wegener’s granulomatosis
